# 18F-FDG PET/CT in xanthogranulomatous cholecystitis with CA199 elevation: diagnostic dilemmas and differentiation strategies

**DOI:** 10.3389/fmed.2025.1630989

**Published:** 2025-09-18

**Authors:** Wanling Qi, Min Chen, Mingyan Shao

**Affiliations:** ^1^Department of Nuclear Medicine, Jiangxi Provincial People’s Hospital, The First Affiliated Hospital of Nanchang Medical College, Nanchang, China; ^2^Department of Pathology, Jiangxi Provincial People’s Hospital, The First Affiliated Hospital of Nanchang Medical College, Nanchang, China

**Keywords:** xanthogranulomatous cholecystitis, gallbladder carcinoma, MRI, CT, 18F-FDG PET/CT

## Abstract

**Objective:**

Xanthogranulomatous cholecystitis (XGC) is a rare and distinctive form of chronic cholecystitis, and it is rather challenging to differentiate it from the thick-walled type of gallbladder carcinoma. Considering that computed tomography (CT), magnetic resonance imaging (MRI), and 18F-fluorodeoxyglucose positron emission tomography/computed tomography (18F-FDG PET/CT) each exhibit unique features in the manifestation of XGC, this study aims to deepen the understanding of XGC and explore the roles of these imaging examinations, especially PET/CT, in the diagnosis and differential diagnosis of XGC.

**Methods:**

We retrospectively analyzed the radiological, clinical, and surgical pathological data of five patients with XGC treated at Jiangxi Provincial People’s Hospital between January 2019 and January 2025.

**Results:**

All five patients with XGC were female, aged 49–84 years. Four patients were hospitalized for recurrent abdominal pain, while one presented with limb weakness. Carbohydrate antigen 19-9 (CA19-9) levels were elevated in three patients. Among the cases in this manuscript, one patient underwent contrast-enhanced MRI, two patients underwent contrast-enhanced CT, and all five patients underwent positron emission PET/CT examination. The results of the imaging examinations revealed that the gallbladder volume was enlarged in three cases and reduced in two cases. The gallbladder wall showed thickening to varying degrees (ranging approximately from 1.2 to 2.0 cm), with diffuse thickening observed in three cases and localized thickening in two cases. The enhancement pattern was characterized by progressive and sustained enhancement, and there was an increase in FDG uptake to different extents. Intramural nodules and gallstones were detected in three cases, and enlarged abdominal or retroperitoneal lymph nodes were found in two cases. The range of the maximum standardized uptake value (SUVmax) was between 7.5 and 19.8, and the median uptake value was 10.04 ± 5.75.

**Conclusion:**

In light of the insufficient diagnostic accuracy of FDG-PET/CT and CA 19-9 in distinguishing XGC from GBC, future efforts should prioritize the validation and adoption of advanced imaging techniques such as FLT-PET/CT. Pending these developments, radical cholecystectomy performed by experienced surgeons remains the recommended surgical strategy for suspected cases.

## 1 Introduction

Xanthogranulomatous cholecystitis (XGC), first reported and defined by McCoy et al. (1), is a rare form of chronic gallbladder inflammation. Thickening of the gallbladder wall may result from malignant conditions such as gallbladder carcinoma (GBC) or from benign entities like XGC (2). Sometimes, it is difficult to differentiate XGC from GBC by the conventional imaging techniques of ultrasonography, computed tomography (CT) and magnetic resonance imaging (MRI). Fluorodeoxyglucose (18F-FDG) positron emission tomography (PET-CT) is useful in differentiating between benign and malignant lesions in the gallbladder. However, 18F-FDG is not specific for malignant lesions, and can accumulate in inflammatory lesions with increased glucose metabolism (3). Few reports about false-positive results on FDG-PET/CT scan (4). We report five cases of irregular gallbladder wall thickening. Among these, three patients exhibited markedly elevated CA19-9 levels. Initial CT or MRI findings suggested GBC in three patients, while FDG-PET scans demonstrated increased metabolic activity in all five cases. However, postoperative pathological examination revealed xanthogranulomatous cholecystitis (XGC) in all cases, with no evidence of malignancy. These findings highlight that contrast-enhanced CT (CECT), MRI, and FDG-PET all led to misdiagnoses of GBC, emphasizing the diagnostic challenge in distinguishing XGC from GBC using conventional imaging modalities. This study aims to improve clinicians’ understanding of this disease through 18F-FDG PET/CT imaging and provide evidence-based guidance for subsequent treatment decisions.

## 2 Methods and materials

### 2.1 Patients and materials

This retrospective study analyzed data from patients with suspected XGC who underwent 18F-FDG PET/CT imaging at Jiangxi Provincial People’s Hospital between January 2019 and January 2025. Initially, a total of eight patients were enrolled. Among them, three were excluded due to lack of pathological confirmation. Consequently, five cases with complete clinical and pathological data were included in the final analysis.

### 2.2 Diagnostic approach

All patients underwent preoperative 18F-FDG PET/CT scans. Imaging of patients was conducted on a PET/CT scanner (GE Healthcare Discovery STE). 18F-FDG with a pH of 5–7 and a radiochemical purity exceeding 95% was produced using a cyclotron (MINItrace, GE Healthcare). All patients underwent fasting for at least 6 h and had blood glucose levels below 7.4 mmol/l. prior to injection with 18F-FDG. Patients were required to lay in a quiet room for 45–60 min after intravenous injection with 5.6 MBq/kg 18F-FDG. Spiral CT scanning was performed at 120 kVp and 300 mA⋅s. PET was performed after spiral CT without patient repositioning. PET images were obtained at 7–8 couch positions per patient, with an acquisition time of 1.5 min per position. We used CT scan data for attenuation correction of PET images and then fused the attenuation-corrected PET and CT images.

The scans were reviewed blindly by one attending physician and one chief physician specializing in PET/CT. In cases of disagreement, a collective decision was made by the departmental physicians. All five patients eventually underwent surgical resection, and the postoperative pathological typing was determined by discussion between one chief pathologist and one attending pathologist.

Informed consent for the anonymous use of clinical data and imaging for publication purposes was provided by all patients. Given the retrospective nature of this study, the institutional review board waived the requirement for approval.

## 3.Results

### 3.1 Case 1

**FIGURE 1:**
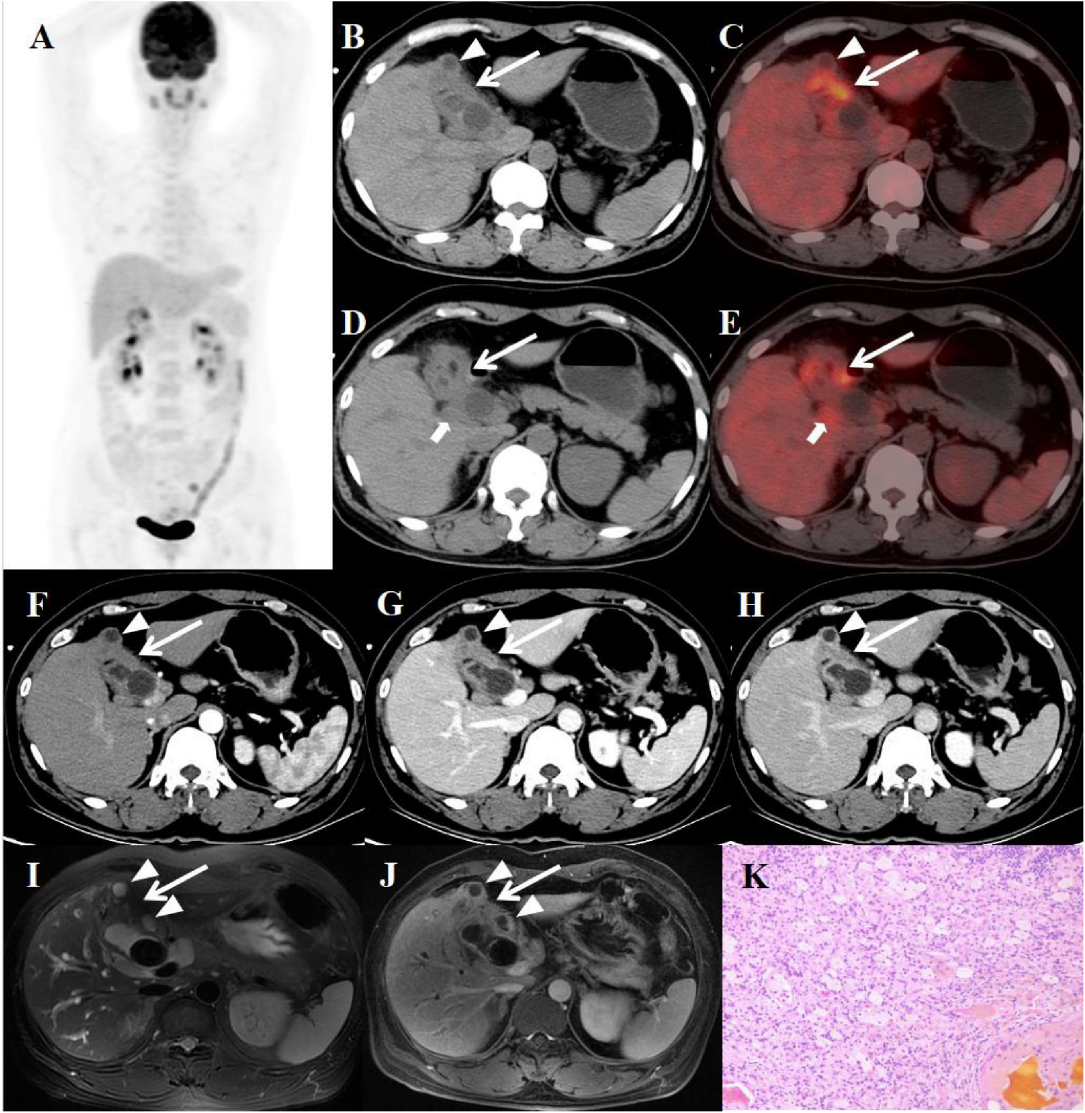
Female, 49 years old. **(A)** (whole body MIP), (**B**–**E**) (Axial CT and fused axial PET-CT), (**F**–**H**) (axial, multiphasic contrast-enhanced CT), **(I)** (axial, MRI-T2WI), **(J)** (axial, enhanced-MRI-delayed phase), and **(K)** (H-E × 40). 18F-FDG PET/CT Imaging Findings: Thickening of the gallbladder wall with indistinct boundaries from the liver, accompanied by increased FDG uptake with a SUVmax of 7.5 (**B**–**E**, arrow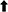). Additionally, several enlarged lymph nodes in the vicinity showed similar heightened FDG uptake, with an SUVmax of 3.5 (**D**, **E**, arrow ↑). The intramural nodules, however, did not display significant FDG uptake (**B**, **C**, arrow ▲). On multiphasic contrast-enhanced CT scans, there was progressive delayed enhancement of the gallbladder wall (**F**–**H**, arrow↑), along with marginal enhancement of the intramural nodules (arrow ↑). The CT values for the non-contrast scan phase, arterial phase, venous phase, and delayed phase were 40, 52, 75, and 89 HU, respectively. The abdominal MRI revealed irregular thickening of the gallbladder wall (I, arrow ↑) accompanied by gallstones. The intramural nodules appeared as hyper-intense on T2WI (arrow ▲). Contrast-enhanced imaging demonstrated delayed enhancement of the gallbladder wall (**J**, arrow ↑), with rim-enhancement of the intramural nodules (arrow ▲). Histopathological analysis confirmed XGC, showing foamy histiocytes intermingled with scattered lymphocytes, encircled by phagocytosed bile, in the absence of malignant cells **(K)**.

A 49-year-old female patient presented with abdominal pain, jaundice, and weight loss for 20 days before admission. An abdominal MRI scan performed at the local hospital disclosed an abnormal thickening of the gallbladder wall, accompanied by intramural nodules and the presence of enlarged pericholedochal lymph nodes. The boundary between the gallbladder and the liver was indistinct, with no evident signs of liver cirrhosis observed. Furthermore, the scan identified multiple stones within the gallbladder, a stone in the lower portion of the common bile duct, and compression of the upper part of the common bile duct, which led to the dilation of both intrahepatic and extrahepatic bile ducts above the obstruction. The multiphase contrast-enhanced scan images revealed that the thickened gallbladder wall shows mild enhancement during the arterial phase, with progressive enhancement observed in the venous and equilibrium phases. The intramural nodules displayed delayed rim enhancement. Despite undergoing treatments with anti-inflammatory and liver-protective agents at the local hospital, her symptoms showed no significant improvement. In response, a percutaneous transhepatic cholangiographic drainage (PTCD) was carried out. To further clarify the diagnosis and treatment plan, the patient was transferred to our hospital, where a comprehensive series of corresponding laboratory tests and imaging studies were conducted. The results of the inflammatory markers indicated that white blood cell count (WBC), neutrophile granulocyte, and C-reactive protein levels were all within normal limits, with procalcitonin was slightly elevated to 0.18 ng/ml. The levels of key tumor markers were as follows: Cancer Antigen 125 (CA-125) was elevated to 60.04 U/ml, while Cancer Antigen 19-9 (CA19-9) was significantly higher at 433.00 U/ml. Due to the patient’s recent PTCD procedure, the follow-up abdominal CT scan showed no evident obstruction or dilation of the bile ducts. The plain scan detected thickening of the gallbladder wall with low-density intramural nodules, surrounding enlarged lymph nodes, and stones in the gallbladder and the distal common bile duct. The enhancement pattern of the lesions on the contrast-enhanced scan was similar to that seen on the MRI. Subsequently, the patient underwent an PET/CT scan to evaluate the systemic condition. The axial CT and fused axial PET-CT images revealed asymmetric thickening of the gallbladder wall, accompanied by elevated fluorodeoxyglucose (FDG) uptake, with a maximum standardized uptake value (SUVmax) of 7.5. Enhanced FDG uptake was observed in several enlarged lymph nodes near the gallbladder fossa, reaching a SUVmax of 3.5. The intramural nodules showed no significant uptake of FDG (Figure 1). Given the imaging findings presented, a suspicion of GBC with hepatic invasion and lymph node metastasis was raised. After discussing the situation with his family, they agreed to proceed with a surgical procedure. The patient subsequently underwent a 5-hour-long procedure, comprising laparoscopic exploration, open partial hepatectomy (anatomical resection of segment IVB and V), choledochotomy, cholecystectomy, bilateral hepaticojejunostomy with Roux-en-Y anastomosis, and lymph node dissection. Considering the possibility of gallbladder cancer invading segment IV of the liver and the porta hepatis, and with no evidence of tumor metastasis in the other abdominal organs, an open abdominal surgical approach was undertaken for treatment. The intraoperative pathology evaluations uniformly pointed to chronic inflammation. Postoperative pathology confirmed the diagnosis of xanthogranulomatous cholecystitis, as well as reactive hyperplasia of regional lymph nodes.

### 3.2 Case 2

**FIGURE 2:**
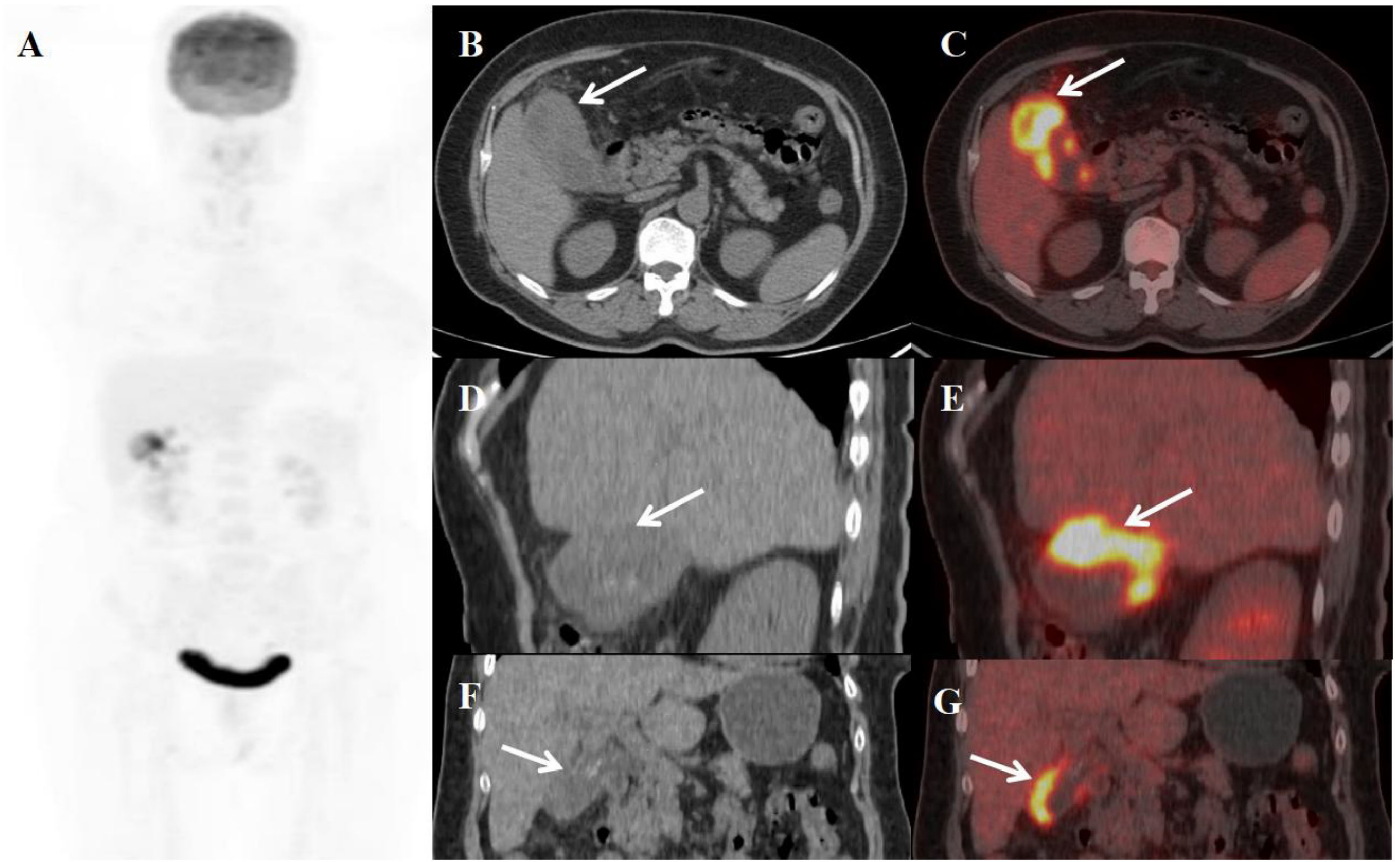
Female, 59 years old. **(A)** (whole body MIP), (**B**, **C**) (Axial CT and fused axial PET-CT), (**D**, **E**) (Coronal CT and fused coronal PET-CT), and (**F**, **G**) (Sagittal CT and fused sagittal PET-CT). 18F-FDG PET/CT Imaging Findings: The gallbladder appears enlarged and adherent, demonstrating diffuse irregular wall thickening (maximum 12 mm) with intramural hypodense nodules (**B**, **D**, **F**, arrow ↑). Significant FDG uptake (SUVmax 19.8) was observed in the lesion, which preserved mucosal continuity despite disruption of the typical hepatobiliary interface (**C**, **E**, **G**, arrow ↑). Accompanying features comprised inflammatory infiltration of pericholecystic adipose tissue and enlargement of retroperitoneal lymph nodes.

A 59-year-old female presented with abdominal pain for two weeks. Physical examination revealed a positive Murphy’s sign. The patient had a history of breast cancer surgery. Initial laboratory findings demonstrated moderate leukocytosis (WBC 12.50 × 10^9^/L) with neutrophilic predominance (81%). Additionally, elevated inflammatory markers were observed, including C-reactive protein (CRP: 13 mg/L) and a mild increase in carbohydrate antigen 19-9 (CA19-9: 70.51 U/mL). All other tumor markers were within normal limits. Abdominal ultrasonography and MRI performed at an outside hospital demonstrated gallbladder wall thickening with multiple intraluminal gallstones. Given the clinical presentation, gallbladder malignancy could not be excluded, prompting referral to our institution for PET/CT evaluation. The PET/CT scan demonstrated an enlarged, adherent gallbladder with diffusely irregular wall thickening (maximum 12 mm) containing intramural hypodense nodules. The lesion showed intense FDG avidity (SUVmax 19.8) while maintaining mucosal integrity, though with loss of the normal hepatobiliary interface. Additional findings included inflammatory changes in pericholecystic fat and enlarged retroperitoneal lymph nodes (Figure 2). These features were initially interpreted as consistent with advanced gallbladder carcinoma with hepatic invasion and nodal metastasis. The patient subsequently underwent definitive surgical management including open cholecystectomy with partial hepatectomy. Final histopathological examination established the diagnosis of XGC, characterized by the hallmark findings of foamy histiocyte aggregates, chronic inflammatory infiltrates, and cholesterol clefts without evidence of malignancy.

### 3.3 Case 3

**FIGURE 3:**
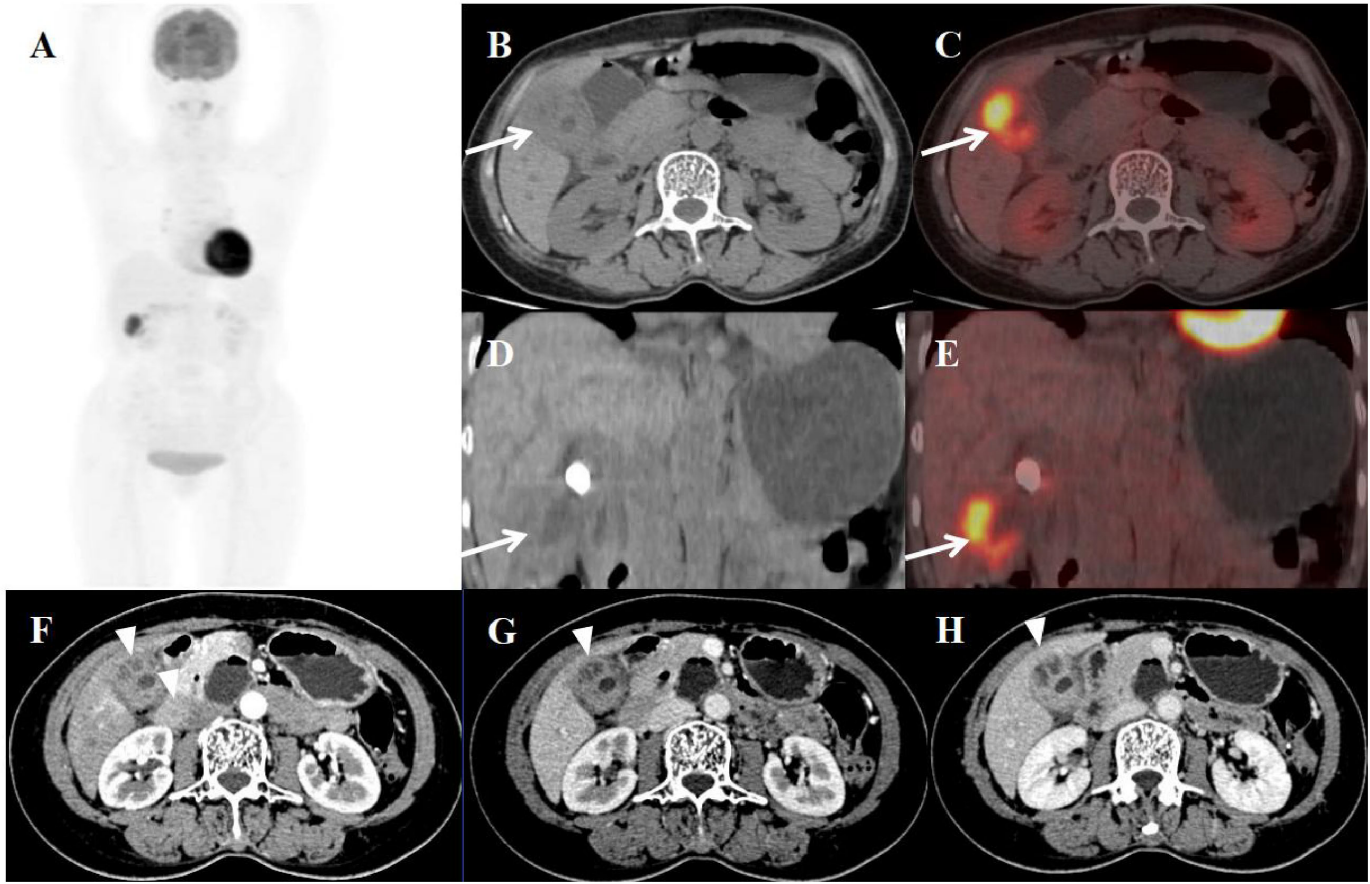
Female, 58 years old. **(A)** (whole body MIP), (**B**, **C**) (Axial CT and fused axial PET-CT), (**D**, **E**) (Coronal CT and fused coronal PET-CT), and (**F**–**H**) (axial, multiphasic contrast-enhanced CT). 18F-FDG PET/CT Imaging Findings: The gallbladder lumen was narrowed with impacted calculi **(D)**. Focal thickening of the gallbladder wall was present, most prominent at the fundus (approximately 20 mm) (**B**, **D**, arrow ↑), accompanied by hypodense intramural nodules. There is markedly increased FDG avidity, with an SUVmax of 10.6 (**C**, **E**, arrow ↑). The hepatobiliary interface is indistinct, though the gallbladder mucosal line remains continuous, and no enlarged lymph nodes are visualized in the surrounding region. Multiphase contrast-enhanced CT reveals progressive delayed enhancement of the thickened gallbladder wall, with ring enhancement of the wall nodules observed in the delayed phase (**H**, arrow ▲) (CT values: 44 HU non-contrast, 67 HU arterial, 76 HU venous, 83 HU delayed).

A 58-year-old woman presented with a three-month history of recurrent right upper abdominal pain and a five-year history of untreated gallbladder stones. Physical examination revealed a positive Murphy’s sign. Laboratory evaluation demonstrated elevated ferritin (355.90 ng/mL), increased C-reactive protein (CRP: 15.54 mg/L), and mildly elevated gamma-glutamyl transferase (49 U/L). All tumor markers were within normal limits. Contrast-enhanced CT demonstrated impacted gallbladder stones with focal wall thickening (maximally 20 mm at the fundus) showing heterogeneous density, progressive persistent enhancement, and internal septations – findings initially suspicious for gallbladder carcinoma with hepatic invasion. Subsequent PET/CT confirmed these features with additional findings of narrowed lumen, intramural hypodense nodules, and markedly increased FDG avidity (SUVmax 10.6), while maintaining mucosal continuity despite an indistinct hepatobiliary interface (Figure 3). The patient required conversion to open cholecystectomy with concomitant partial hepatectomy due to extensive inflammatory adhesions. Histopathological examination of the surgical specimen established the definitive diagnosis of xanthogranulomatous cholecystitis (XGC), demonstrating the characteristic histologic triad of: (1) foamy histiocytic aggregates, (2) chronic inflammatory infiltrates, and (3) fibroblastic proliferation with cholesterol clefts.

### 3.4 Case 4

**FIGURE 4:**
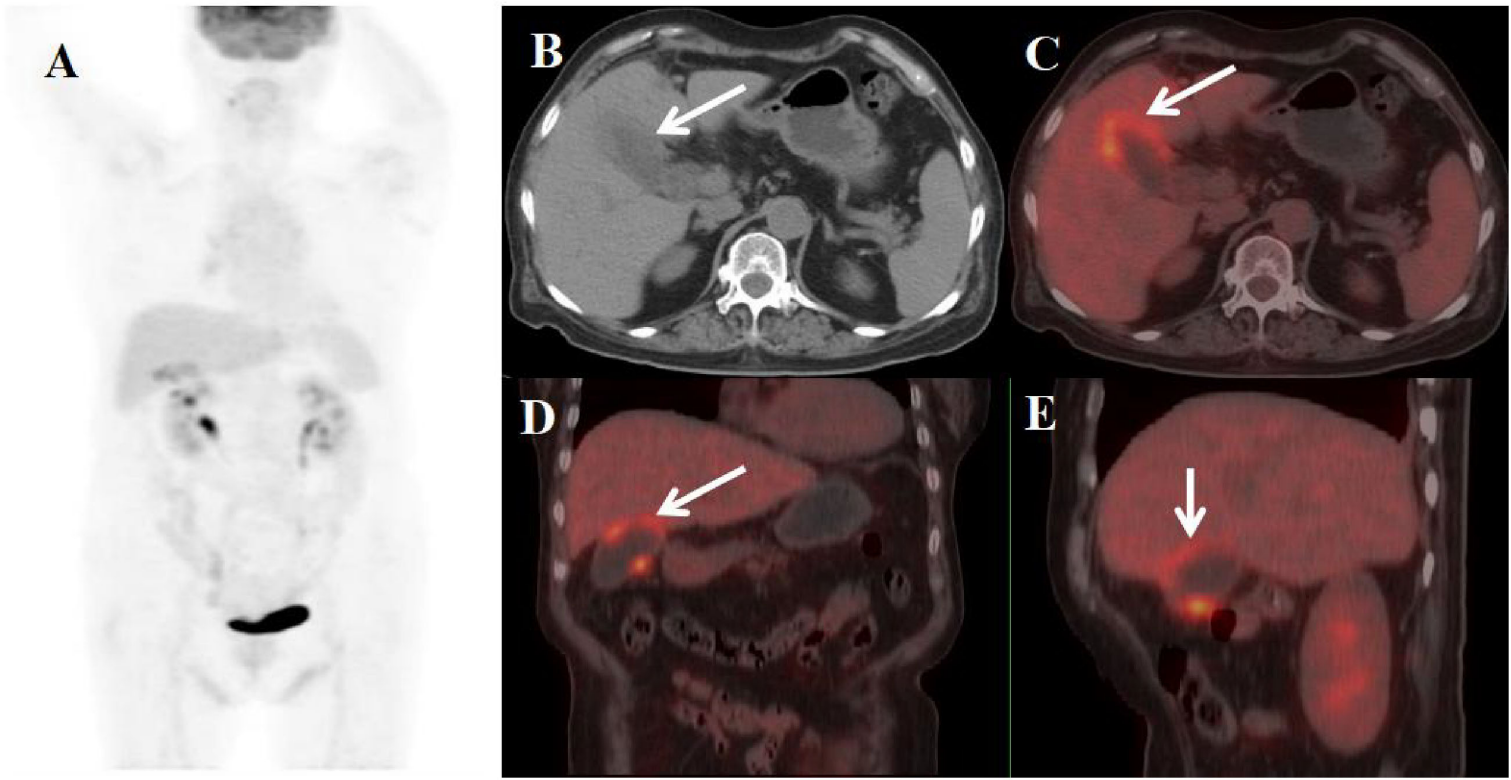
Female, 70 years old. **(A)** (whole body MIP), (**B**, **C**) (Axial CT and fused axial PET-CT), **(D)** (Fused coronal PET-CT), and **(E)** (Fused sagittal PET-CT). 18F-FDG PET/CT Imaging Findings: The gallbladder demonstrates focal mural thickening (maximum 12 mm) with overall enlargement (**B**, **C**, arrow ↑), while maintaining mucosal integrity and a distinct hepatobiliary interface. These morphological changes are accompanied by localized FDG avidity (SUVmax 5.8) (**D**, **E**, arrow ↑).

A 70-year-old woman presented with generalized limb weakness persisting for over one month. She had a history of long-standing hypertension (>20 years), recently diagnosed diabetes mellitus (1 year duration), and chronic hypothyroidism (>10 years). Initial laboratory evaluation showed normal white blood cell count (WBC 4.5-10 × 10^9^/L), neutrophil percentage (40–75%), and C-reactive protein level (CRP < 5 mg/L), but demonstrated markedly elevated tumor markers: carbohydrate antigen 19-9 (CA19-9 362.00 U/mL; reference < 27) and carbohydrate antigen 125 (CA125 130.00 U/mL; reference < 35). The patient was subsequently referred for PET/CT evaluation, which demonstrated: (1) gallbladder enlargement with focal wall thickening (maximum 12 mm), (2) preserved mucosal continuity, (3) clear liver-gallbladder interface, and (4) focally increased FDG uptake (SUVmax 5.8) (Figure 4). Following laparoscopic cholecystectomy, histopathological examination confirmed XGC, characterized by foamy histiocyte infiltration and chronic inflammatory changes.

### 3.5 Case 5

**FIGURE 5:**
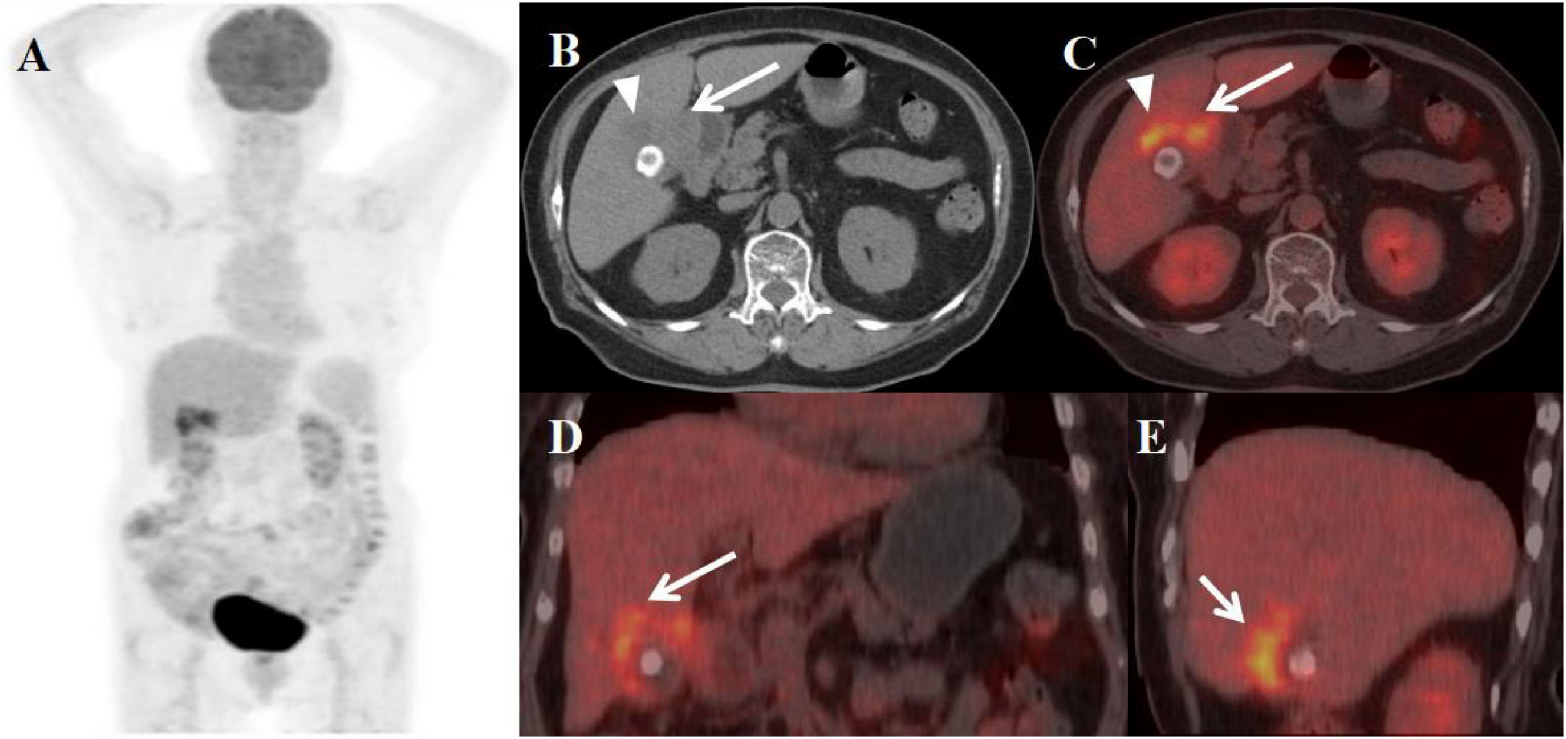
Female, 84 years old. **(A)** (whole body MIP), (**B**, **C**) (Axial CT and fused axial PET-CT), **(D)** (Fused coronal PET-CT), and **(E)** (Fused sagittal PET-CT). 18F-FDG PET/CT Imaging Findings: The gallbladder demonstrates diffuse wall thickening (maximum 1.9 cm) with luminal narrowing (**A**, arrow ↑), while maintaining mucosal integrity. The hepatobiliary interface appears indistinct, with a 2.3 cm mildly hypodense hepatic lesion noted adjacent to the gallbladder (**B**, arrow ▲). The thickened gallbladder wall shows duodenal bulb adhesion, with both the mural thickening and adjacent hepatic lesion exhibiting moderate FDG avidity (SUVmax 6.5). Associated cholelithiasis is present.

An 84-year-old woman presented with a two-day history of abdominal pain. Physical examination revealed a positive Murphy’s sign. Laboratory evaluation revealed leukocytosis (WBC count: 11.43 × 10^9^/L) with neutrophil predominance (85%). CRP was elevated (15 mg/L). All tumor markers were within normal limits. Since the possibility of malignant gallbladder lesions could not be excluded, the patient underwent PET/CT in our department. The imaging revealed a constricted gallbladder lumen with diffuse wall thickening, measuring up to 1.9 cm at its maximum. The mucosal line remained intact. The liver-gallbladder interface appeared blurred, with a round, mildly hypodense lesion (approximately 2.3 cm in diameter) observed in the adjacent hepatic parenchyma. Furthermore, the thickened gallbladder wall demonstrated adhesion to the duodenal bulb. These lesions exhibited increased FDG avidity, with a SUVmax of 6.5 (Figure 5). Concurrent gallbladder stones were also identified. The patient subsequently underwent cholecystectomy with partial resection of liver segment V and adhesiolysis. Postoperative histopathological examination confirmed the diagnosis of XGC with concurrent hepatic abscess.

### 3.6 Findings summary

All five patients with XGC were female, ranging in age from 49 to 84 years (median age: 64 years). The duration of symptoms varied from 2 days to 3 months. Four patients were hospitalized due to recurrent abdominal pain, and three exhibited a positive Murphy’s sign. Laboratory findings showed elevated WBC counts and neutrophil percentages in two patients. Additionally, CRP levels were increased in three patients, and CA19-9 levels were elevated in three patients as well. Imaging studies revealed gallbladder enlargement in three patients and reduced volume in two. The gallbladder wall was thickened to varying degrees, ranging from approximately 1.2–2.0 cm. Diffuse thickening (involving > 50% of the gallbladder wall circumference) was observed in three patients, while two had localized thickening (involving ≤ 50%). The mucosal line remained intact in all five cases, and intramural nodules were detected in three. The liver-gallbladder boundary was indistinct in four patients but clearly defined in one. One patient presented with a concurrent liver abscess and duodenal bulb invasion. Gallbladder stones were identified in three patients, and enlarged abdominal or retroperitoneal lymph nodes were found in two. Two patients exhibited persistent enhancement on contrast-enhanced CT scans. FDG PET/CT imaging demonstrated increased metabolic activity in all cases, with a SUVmaxranging from 7.5 to 19.8 (median: 10.04 ± 5.75). Preoperatively, all patients were misdiagnosed with gallbladder carcinoma; however, postoperative pathological examination confirmed XGC. The clinical, surgical, and imaging findings of these patients were summarized in Tables 1–3, respectively.

**TABLE 1:** Clinical manifestations of xanthogranulomatous cholecystitis.

Parameter	Case 1	Case 2	Case 3	Case 4	Case 5
**Demographics**					
Gender	Female	Female	Female	Female	Female
Age (years)	49	59	58	70	84
**Clinical features**					
Abdominal pain	Yes	Yes	Yes	No	Yes
Murphy’s sign	No	Yes	Yes	No	Yes
**Laboratory findings**					
WBC (× 10^9^/L)	6.24	12.50	3.68	4.50	11.43
Neutrophils (× 10^9^/L)	4.06	10.13	1.58	3.20	9.72
CRP (mg/L)	3.22	13.00	15.54	4.10	15.00
CA19-9 (U/ml)	433.00	70.51	26.02	362.00	19.15

Note: Reference ranges were defined according to standardized laboratory medicine guidelines and our institutional clinical laboratory (5): White blood cell count (WBC): 4.0−20.0 × 10^9^/L; Neutrophils: 2.0−7.0 × 10^9^/L; C-reactive protein (CRP): <8.0 mg/L; Carbohydrate antigen 19-9 (CA19-9): <37.0 U/mL.

**TABLE 2:** Surgical data of xanthogranulomatous cholecystitis.

Parameter	Case 1	Case 2	Case 3	Case 4	Case 5
**Treatment**					
Treatment method	Surgery	Surgery	Surgery	Surgery	Surgery
**Operative details**					
Surgical team	Standard HPB Team*	Standard HPB Team*	Standard HPB Team*	Standard HPB Team*	Standard HPB Team*
Operative time (hours)	5	4	3	1	4
Blood loss (mL)	300	200	160	80	250
**Postoperative outcomes**					
Hospital stay (days)	11	10	7	5	15
Complications	None	None	None	None	Fear
Follow-up (recurrence)	None	None	None	None	None

Note: *All procedures were performed by a dedicated hepatobiliary (HPB) surgical team. The core team included an attending or above HPB specialist surgeon (as the primary operator), an assistant surgeon, and a scrub nurse proficient in advanced laparoscopic and open instrumentation. For cases with anticipated complexity (e.g., severe inflammation), the team was augmented with a second assistant.

**TABLE 3:** Imaging manifestations of xanthogranulomatous cholecystitis.

Parameter	Case 1	Case 2	Case 3	Case 4	Case 5
Volume	Enlarged	Enlarged	Reduced	Enlarged	Reduced
Wall thickening	Diffuse	Diffuse	Focal	Focal	Diffuse
Intramural nodules	Yes	Yes	Yes	No	No
Cholelithiasis	Yes	Yes	Yes	No	Yes
Mucosal lining	Continuous	Continuous	Continuous	Continuous	Continuous
Liver-GB border	Blurred	Blurred	Blurred	Clear	Blurred
Lymphadenopathy	Yes	Yes	No	No	No
SUVmax	7.5	19.8	10.6	5.8	6.5

### 3.7 Treatment and follow-up

Case 1: The patient was discharged on postoperative day 11. The patient’s preoperative CA19-9 levels were significantly elevated at 433.00 U/ml. Subsequent to the surgical procedure, a three-month follow-up examination indicated that the CA19-9 levels had reverted to normal at 14.15 U/ml.

Case 2: The postoperative course was uneventful and the patient was discharged on postoperative day 10. Follow-up CT imaging at two months postoperatively showed no abnormalities, with complete normalization of serum CA19-9 (15 U/mL). The patient remains disease-free and in good health three years after surgery.

Case 3: The patient underwent suture removal on the 7th postoperative day and was subsequently discharged from the hospital. A clinical and radiological follow-up at one month postoperatively showed no abnormalities.

Case 4: Two-month postoperative follow-up CT demonstrated complete resolution of previous abnormalities, with serum tumor markers CA19-9 and CA12-5 normalizing to 21.25 and 5.0 U/mL respectively.

Case 5: The patient was subsequently discharged from the hospital. The patient achieved successful discharge on postoperative day 15. Surveillance imaging performed at 2-month follow-up showed no recurrent lesions.

## 4 Discussion

Xanthogranulomatous cholecystitis (XGC) is characterized by destructive inflammation, intramural nodules with infiltration of foamy histiocytes and macrophages, and proliferative fibrosis (6). The prevalence ranges from 1 to 9% (7). It usually occurs in the population older than 50 years and has no gender predominance (8). Clinical manifestations of XGC are non-specific, including right upper abdominal pain, nausea, vomiting, obstructive jaundice, palpable mass, or positive Murphy’s sign (9). The mechanism of formation of XGC has not yet been clearly elucidated; however, the preliminary cause is considered to be the extravasation of bile into the gallbladder wall as a result of the involvement of the Rokitansky-Aschoff sinuses or the progression of a small mucosal ulcer. The presence of gallstones or a biliary obstruction plays an important role in the development of XGC (10). Clinical evaluation of the five patients revealed cholelithiasis in four cases (80%) and biliary tract obstruction in one case (20%). In addition, despite its benign character, XGC shows locally aggressive features with various complications, such as perforation, abscess, and fistula formation, and often extends to adjacent organs which including the liver, duodenum, and, frequently the hepatic flexure colon (9). In the present study, one case demonstrated concurrent liver abscess formation and duodenal involvement. Owing to these aggressive features, the radiologic features of XGC overlap with wall-thickening type GBC, and the differential diagnosis of the two diseases often remains challenging.

In clinical practice, some patients with XGC are misdiagnosed as having GBC, leading to unnecessary radical surgery or being incorrectly informed as having advanced GBC (11, 12). In these five cases, GBC was strongly suspected before surgery based on laboratory and imaging findings. First, elevated CA19-9 levels were observed in three patients. Although gallstones were identified in these cases, no clinical evidence of inflammation (such as fever or elevated inflammatory markers) was present. Second, cross-sectional imaging (CT/MRI) demonstrated significant irregular gallbladder wall thickening in selected cases, accompanied by adjacent liver parenchymal infiltration. Regional lymphadenopathy was variably present. Third, FDG-PET imaging revealed lesions with markedly increased metabolic activity. Given the high preoperative suspicion of GBC, extended cholecystectomy with regional lymphadenectomy was performed as the appropriate surgical management. More extensive and invasive surgery, such as the total resection of the extrahepatic bile duct or a pancreatoduodenectomy, would have been appropriate if the intraoperative pathological examination of the lymph nodes had been malignant. Fortunately, the pathologicaldiagnosis from paraffin sections of the GB wall after surgery revealed only XGC without malignancy.

Physical examination and laboratory findings are not sufficient to distinguish XGC from GBC. Tumor markers such as CEA and CA19-9 lack specificity as they can be elevated or within the normal ranges in patients with either XGC or GBC. Elevated CA19-9 in XGC maybe caused by inflammation-induced bile duct damage, resulting in increased secretion of CA19-9 by epithelial cells (13). Conversely, some GBC patients may have normal tumormarker levels. However, tumor markers can be used for postoperative follow-up monitoring (14). The levels of tumor markers in XGC patients may decrease after surgery, whereas they remain elevated in GBC patients, offering a relatively reliable means of identification (15). In this manuscript, the CA19-9 levels were elevated preoperatively in three patients. Upon reexamination 1–3 month after the surgery, the CA19-9 levels of these patients had returned to the normal range.

In clinical practice, abdominal ultrasound (US), CT, and MRI are the commonly used imaging modalities for the assessment of gallbladder disease. The common imaging findings of XGC from CT include the diffuse type of gallbladder wall thickening (range > 50% gallbladder circumference), the co-existence of gallstones, intramural nodules, a continuous mucosal line (7, 12, 16). The inflammatory change of XGC appears in the Rokitansky-Ashoff sinuses, not in the mucosa, resulting in full-thickness involvement of the gallbladder wall. Therefore, the XGC shows diffuse gallbladder wall thickening and mucosal uniformity (17). Additionally, the signal drops seen on chemical shift gradient-echo MR imaging, and T2-hyperintense, delayed-enhancing intramural nodules have come to be regarded as characteristic findings of XGC (18, 19). Microscopically, these intramural nodules demonstrate xanthogranulomatous infiltrates, abscesses, necrotic areas, or combinations thereof (20). In the present study, intramural nodules were identified in three cases; nevertheless, initial diagnostic errors occurred due to insufficient clinical experience with such pathological features. Conversely, limited gallbladder wall thickening, discontinuity of the enhancing mucosal line and gallbladder mucosal retraction were observed in patients with wall-thickening type GBC (21). Besides, GBC showed earlier peak enhancement than XGC in a dynamic contrast-enhanced study. This is because malignant tumor growth is accompanied by angiogenesis, while inflammation increases blood flow without angiogenesis (22). However, the other imaging findings including regional lymph node enlargement, pericholecystic infiltration, and adjacent organ invasion were similar between XGC and the wall-thickening type of GBC (23).

FDG-PET is a valuable imaging approach for differentiating between benign and malignant lesions in the gallbladder. However, there is a limitation in the ability of FDG-PET to differentiate between inflammatory and malignant lesions. It is true that the tracer can accumulate in inflammatory lesions with increased glucose metabolism, false-positive results have been obtained with benign lesions, including chronic cholecystitis, adenomyomatosis of the gallbladder (4). In dual-phase imaging, if the delayed SUVmax increases by more than 10% compared to the initial imaging, it is more indicative of a malignant tumor diagnosis. Conversely, if the increase is less than 10%, it tends to suggest a benign lesion (3). In our patient, FDG-PET CT scans showed increased activity in the thickened wall of the gallbladder which was associated with an inflammatory, not malignant, lesion, without the performance of a delayed imaging study. Notably, Case 1 demonstrated complete absence of FDG avidity in the intramural nodule, correlating histopathologically with abscess necrosis. Conversely, Cases 2 and 3 exhibited intense FDG uptake within their intramural nodules, corresponding to xanthogranulomatous inflammation on pathological examination. Therefore, FDG-PET may used more frequently to diagnose GBC, providing a comprehensive view of the entire body to aid in staging the disease (24). In contrast, 18F-fluorothymidine (FL-T) overcomes this imaging limitation and is extensively studied as an imaging agent for assessing tumor cell proliferation. The diagnostic accuracy of FL-T-PET/CT in differentiating benign and malignant biliary tumors is 92%, which is superior to the accuracy of FDG-PET/CT and contrast-enhanced CECT methods. It has potential to prevent avoidable radical surgery in 33.3% cases of resectable pancreatobiliary tumors (25).

Despite continuous advancements in diagnostic imaging technology for gallbladder lesions, histopathology remains the gold standard for diagnosing these conditions. Endoscopic ultrasonography (EUS) combined with fine-needle aspiration (EUS-FNA) is useful for distinguishing between benign and malignant gallbladder lesions (26). The treatment for XGC is simple laparoscopic cholecystectomy (LC), whereas GBC may require wider excision and dissection of regional lymph nodes (27). A recently published systematic review indicated that approximately half of all XGC cases necessitated open cholecystectomy due to severe adhesions and extensive local inflammatory infiltration, with a conversion rate approaching 35%. Despite the technical complexity associated with these procedures, both mortality and complication rates remain low, reported at 0.3% and 2–6%, respectively (27). Therefore, exact preoperative diagnosis is vital for appropriate surgery planning. In this study, for select cases where laparoscopic exploration raised suspicion of malignant gallbladder carcinoma with hepatic invasion, the surgical approach was converted to open radical cholecystectomy. Histopathological examination of surgical specimens revealed foam cell infiltration without evidence of malignant cells, ultimately confirming a diagnosis of XGC.

In summary, XGC closely mimics GBC in clinical, laboratory, and imaging features, often leading to misdiagnosis and potential overtreatment. Key discriminators include intramural nodules, continuous mucosal lining on cross-sectional imaging, and transient CA19-9 elevation that normalizes postoperatively; however, due to significant overlap, histopathological confirmation remains essential. Preoperative recognition of these characteristics-coupled with intraoperative frozen section when malignancy is suspected-can guide appropriate surgical planning, avoiding unnecessarily radical procedures while ensuring complete treatment.

## 5 Conclusion

This study confirms that FDG-PET/CT and CA 19-9 demonstrate limited accuracy in distinguishing XGC from GBC, as evidenced by preoperative misdiagnosis in all five presented cases. These findings underscore the critical need for advanced diagnostic modalities such as FLT-PET/CT to improve preoperative differentiation. Until these novel techniques become clinically viable, radical cholecystectomy performed by experienced hepatobiliary surgeons remains the recommended approach when malignant transformation cannot be definitively excluded based on current imaging and serological markers.

## Data Availability

The original contributions presented in this study are included in this article/supplementary material, further inquiries can be directed to the corresponding authors.
